# Presence of anti-cyclic citrullinated peptide antibody isotypes in juvenile idiopathic arthritis synovial fluid indicates autoantibody production at the site of inflammation

**DOI:** 10.1186/1546-0096-10-S1-A118

**Published:** 2012-07-13

**Authors:** Brooke E Gilliam, Sandra Crespo-Pagnussat, Judy Ko, Reema H Syed, Terry L Moore

**Affiliations:** 1 St. Louis University, St. Louis, MO, USA

## Purpose

To determine the presence and significance of anti-cyclic citrullinated (anti-CCP) antibody isotypes (IgA, IgG, IgM, and IgA/IgG) in the synovial fluid (SF) of juvenile idiopathic arthritis (JIA) patients.

## Methods

Anti-CCP antibody isotypes (IgA, IgG, IgM, and IgA/IgG) were assayed by enzyme-linked immunosorbent assays in the SF of 47 individual JIA patients. Patients included 28 oligoarthritis, 11 IgM RF- polyarthritis, 3 IgM RF+ polyarthritis, 3 enthesitis-related, 1 psoriatic, and 1 systemic-onset. As non-inflammatory controls, 10 patients aspirated SF with osteoarthritis (OA) were used.

## Results

Eleven of 47 (23%) JIA SF samples were positive for the different anti-CCP antibody isotypes. IgM anti-CCP antibodies were positive in 9 JIA patients, including 5 oligoarthritis, 3 IgM RF+ and 1 IgM RF- polyarthritis. IgG anti-CCP antibodies were positive in 8 JIA patients, including 4 oligoarthritis, 2 IgM RF+, 1 IgM RF- polyarthritis, and 1 enthesitis-related. Two IgM RF+ polyarthritis patients were positive for IgA anti-CCP antibodies, and also positive for all 3 isotypes. Four JIA patients were positive for 2 anti-CCP antibody isotypes, including 1 IgM RF- polyarthritis patient (IgG and IgM) and 3 oligoarthritis patients (2 IgG and IgM, 1 IgA and IgM). Five JIA patients were positive for 1 anti-CCP antibody isotype, including 3 oligoarthritis (2 IgM, 1 IgG), 1 enthesitis-related (IgG), and 1 IgM RF+ polyarthritis patient (IgM). Three JIA patients were positive for the combined IgA/IgG anti-CCP antibody ELISA, including 2 with IgM RF+ polyarthritis and 1 enthesitis-related. Polyarthritis patients demonstrated significantly higher levels of all anti-CCP antibody isotypes compared to the other JIA subtypes (p<0.05). When only evaluating the IgM RF+ polyarthritis patients against the other subtypes, the levels were even more significantly increased (p<0.05). Serum-matched IgG anti-CCP antibody data was available in 27 JIA patients and 5 healthy controls. All 3 IgM RF+ polyarthritis patients were positive for the IgG anti-CCP antibodies in serum and SF. Two JIA patients who were positive for IgG anti-CCP antibodies in SF had a negative result in serum. Date-matched SF and serum IgM and IgA data was available on 12 JIA patients. More JIA patients were positive for IgA anti-CCP antibodies in serum compared to SF, while SF showed increased positivity for IgM anti-CCP antibodies compared to serum.

## Conclusion

While IgG anti-CCP antibodies are found in JIA patients with polyarticular disease, we found that IgM and IgG anti-CCP antibodies were detected in the SF of several subtypes of JIA. The most commonly found anti-CCP antibody isotype in JIA SF was IgM, which may partly be indicative of defective B cell class switching in these patients. IgM also plays a significant role in complement activation, indicating the significance of the complement system in JIA pathogenesis. Measurement of only IgG anti-CCP antibodies would have overlooked 4 JIA patients with elevated levels of IgA and/or IgM anti-CCP antibodies in SF, indicating the importance of measuring all 3 isotypes in SF.

## Disclosure

Brooke E. Gilliam: None; Sandra Crespo-Pagnussat: None; Judy Ko: None; Reema H. Syed: None; Terry L. Moore: None.

